# Mapping the Scientific Research on Suicide and Physical Activity: A Bibliometric Analysis

**DOI:** 10.3390/ijerph192416413

**Published:** 2022-12-07

**Authors:** Ángel Denche-Zamorano, Damián Pereira-Payo, Juan Manuel Franco-García, Raquel Pastor-Cisneros, Guido Salazar-Sepúlveda, Dante Castillo, Miseldra Marín-Gil, Sabina Barrios-Fernandez

**Affiliations:** 1 Promoting a Healthy Society Research Group (PHeSO), Faculty of Sport Sciences, University of Extremadura, 10003 Caceres, Spain; 2 Health, Economy, Motricity and Education Research Group (HEME), Faculty of Sport Sciences, University of Extremadura, 10003 Cáceres, Spain; 3 Departamento de Ingeniería Industrial, Facultad de Ingeniería, Universidad Católica de la Santísima Concepción, Concepción 4090541, Chile; 4 Facultad de Ingeniería y Negocios, Universidad de Las Américas, Concepción 4090940, Chile; 5 Centro de Estudios e Investigación Enzo Faletto, Universidad de Santiago de Chile, Santiago 9170022, Chile; 6 Public Policy Observatory, Universidad Autónoma de Chile, Santiago 7500912, Chile; 7 Occupation, Participation, Sustainability and Quality of Life (Ability Research Group), Nursing and Occupational Therapy College, University of Extremadura, 10003 Cáceres, Spain

**Keywords:** scientometrics, suicide, mental health, physical exercise

## Abstract

This research provides an overview of the current state of scientific literature related to suicide and physical activity (PA). A bibliometric analysis of studies published between 1996 and 2022 in The Web of Science (WoS) was carried out, applying the traditional bibliometric laws, using Microsoft Excel and the VOSviewer software for data and metadata processing. A total of 368 documents (349 primary research and 19 reviews) were extracted from 70 WoS categories. The results revealed an exponential increase in scientific production from 2017 to 2022 (R^2^ = 88%), revealing the United States hegemony being the most productive country, with 156 of the publications (42.4%), the most cited (4181 citations) being the centre of a collaborative network with links to 35 countries and having April Smith, from the Miami University, as the most prolific author (eight publications) and Thomas Joiner, from the Florida State University, as the most cited author (513 citations). The Psychiatry WoS category, with 155 papers, had the highest number of publications, and The Journal of Affective Disorders, from Elsevier, had the highest number of published papers within this category.

## 1. Introduction

The term suicide encompasses a broad group of behaviours including thoughts, ideas, gestures, planning, attempts, completions, or others [1]. Thus, suicidal ideation describes a range of contemplations, wishes, and preoccupations with death and suicide [2]; suicide attempt is any self-directed and potentially harmful action with the intent to die even if the behaviour does not result in injury; and suicide is usually used to define the death caused by self-directed injurious behaviour [3]. Some warning signs for suicidal behaviours are suicidal thoughts and nonfatal suicide attempts [4]. Both suicide ideation and attempted suicide strongly predict suicide deaths and have negative consequences such as injury, hospitalization, and loss of liberty, which entails large economic costs for public institutions [4,5]. 

Suicide, classified as an external cause of death by the World Health Organization (WHO) [6], is a leading cause of death and disability worldwide and is a matter of concern worldwide: 700,000 people die due to this cause every year, placing it as the third leading cause of lost life-years [7]. Europe has been reported to have the highest suicide rate per 100,000 person-year, followed by Asia, Africa, North America, and Oceania [8]. Sex disparities exist, as figures show that women have an increased risk to attempt suicide compared to men, but men’s suicide rate is three times higher [9]. Among the general population, suicidal ideation prevalence is estimated to be about 9.2%, whereas suicide attempts are considered approximately 2.7% [5]; in the population with major depressive disorder, the suicide attempts prevalence increases to 31% [10]. 

Life satisfaction, which is the individual’s quality of life according to his/her perception [11], is a strong predictor of mortality and psychiatric morbidity [12] and has a long-term effect on suicide risk [12]. Self-esteem is associated with total self-esteem, social self-esteem, and family self-esteem [13]: people who attempt suicide usually have lower self-esteem than the general population and psychiatric patients with no previous suicidal behaviours [14,15,16]. Adequate levels of perceived social support, i.e., the individual’s perception of the presence of his/her family, friends, and other people that supply psychological and tangible endorsement [17], were shown to have a protective role in young adults [18], university students [19], and adults [20]. Living through conflicts, catastrophes, violent acts, abuse, the loss of loved ones, and feelings of isolation are risk factors that can lead to suicidal behaviours [7]. Owning a firearm was found to be a risk factor in the general population [21,22]. In addition, living in low- and middle-income countries is a factor since 77% of deaths occur in those countries [7]. Furthermore, suicidal ideation, hopelessness, alcohol dependence, alcohol abuse, a low level of social and occupational functioning, and poor perceived social support were found to significantly predict suicide attempts [23]. Nevertheless, death by suicide is higher among those who have attempted it in the past [7,24].

All the above highlights two key aspects of suicide prevention and treatment: (1) the multi-causality [25,26] and (2) the great weight of social factors [27,28] such as age, marital status, the existence or not of social relationships and a support network, having suffered or lived stressful life situations, as well as addictions and the state of physical (pain) and mental health. A person does not attempt suicide for a single cause but because of the interaction of a series of risk, protective, and precipitating factors [3,29,30]. Suicide prevention and treatment require an interdisciplinary approach [31,32,33], encompassing population education, support/assistance services, and diverse treatment services for the most vulnerable people, those at some point on the suicide spectrum, survivors, and their families.

Physical activity (PA) is any bodily movement produced by skeletal muscles that require energy expenditure [34] and is related to different health benefits including better weight control [35], pain management [36,37], and psychiatric symptomatology [38], among many others [39]. Some studies support that not meeting PA guidelines is associated with increased suicidal ideation and that PA could be a promising method for reducing it [40,41,42] and suicide attempts [43]. Moreover, promoting PA and positive mental health may be a relevant strategy in the prevention of suicidal ideation and behaviour [44]. The WHO recommends performing 75–150 min minutes of vigorous-intensity aerobic PA or an equivalent combination for substantial health benefits [45]. Modifiable health factors play an important role in mental health: there are associations between low PA levels and unhealthy and sedentary behaviours although with sex differences. One study found that feelings of sadness and hopelessness were associated with not meeting the aerobic PA guidelines (only boys), not playing in at least one sports team, and using the computer for more than two hours a day; suicidal thoughts with not meeting the aerobic PA guidelines and using the computer more than two hours a day; and suicide attempts with watching TV more than two hours a day and playing video/computer games more than two hours a day (men) [46]. However, among a sample of 136,857 adolescents from 48 countries, meeting PA guidelines was associated with lower odds for suicide attempts in boys but higher odds for suicide attempts in girls, so future research should maintain a sex perspective in this regard [47]; although more research is needed, it is hypothesised that body dissatisfaction and low self-esteem may be driving extrinsic motivations for PA practice. 

Scientific mappings, through descriptive bibliometric statistical studies, quantitatively analyse the existing scientific publications on a topic, providing relevant information such as the interest of the topic in the scientific field, the trend followed by annual publications, the most productive and most relevant researchers, the most involved journals with the most cited articles, the most co-authored countries, the most used keywords, the most cited articles, and other information that facilitates understanding of the state of the art in a specific topic [48,49]. This information may be beneficial both for authors, who can learn about the journals involved in the topic, but also to other authors or research groups working on the topic, favouring the establishment of potential collaborations between researchers from different institutions and countries, as well as for journals and publishers to understand the research interest related to the topic and the number and profile of researchers concerned with this research topic [50]. Although there are bibliometrics on topics related to suicide [51,52,53,54], there have been none found in publications related to suicide and PA. Since PA is considered a protective factor for mental health [46,47], this study aimed to analyse the exponential growth of annual publications on suicide and AP, identifying the most relevant, used, and cited authors, keywords, papers, and journals in this field.

## 2. Materials and Methods

### 2.1. Design and Data

This study offers a descriptive bibliometric analysis of scientific literature on suicide and AP published in journals indexed in The Web of Science (WoS), one of the most widely used databases by researchers in bibliometric analysis, due to the broad range of information available and the quality of the journals that are indexed.

### 2.2. Data Collection and Search Strategy

Bibliometric data were retrieved by conducting a search on 20 June 2022 in The WoS Core Collection, including the editions Science Citation Index Expanded (SCI-EXPANDED) and Social Sciences Citation Index (SSCI). The search vector used was Ti = (suicid*) AND (Ts = (“Physical activity”) OR Ts = (exercise) OR Ts = (sport*) OR Ts = (“Physical inactivity”) OR Ts = (sedentary)); where Ti represents the tag for searching the paper titles and Ts for searching the topic (title, abstract, author keywords, and keyword Plus^®^). Primary research and reviews were included, excluding editorial materials, meeting abstracts, letters, book reviews, corrections, and news items. 

### 2.3. Data Analysis

A descriptive analysis of the annual publications was carried out, verifying their trend and whether they were in an exponential growth phase analysed with the coefficient of determination (R^2^), using DeSolla Price’s law of exponential growth of science [55,56,57]. An analysis of the categories into which the set of documents was assigned in WoS was carried out. Datasets were downloaded in plain text for further analysis and visualisation in Microsoft^®^ Excel^®^ for Microsoft 365 MSO version 2206 and the VoSViewer software. Bradford’s law of dispersion of science was applied to identify the core journals with the most published and cited papers on the topic [58]. A search and removal of duplicate co-authors were carried out. Lotka’s law [59] was applied to determine the most prolific authors, and based on this, the Hirch index (h-index) [60] was used to identify the most prominent authors (n authors with at least n citations). Publications were analysed by country, offering interrelation graphs between them. The most cited publications were selected by applying the h-index considering as the most relevant articles the h articles with at least h citations [48]. The keywords most used by the authors were defined by applying Zipf’s law [61]. Interrelation graphs were created with WOSviewer for co-authors and the most prolific and highly cited journals, countries, and author keywords as well as co-citations.

## 3. Results

### 3.1. WoS Categories

A total of 368 documents (349 primary research and 19 reviews) were extracted from the WoS categories. Table 1 shows the WoS top 10 categories according to the number of publications, the journal with the most publications in each category, and the publisher it belongs to. The Psychiatry category, with 155 papers, had the highest number of publications, and The Journal of Affective Disorders (22), from Elsevier, had the highest number of papers published in this category.

### 3.2. Annual Publications Trend

**Figure 1:**
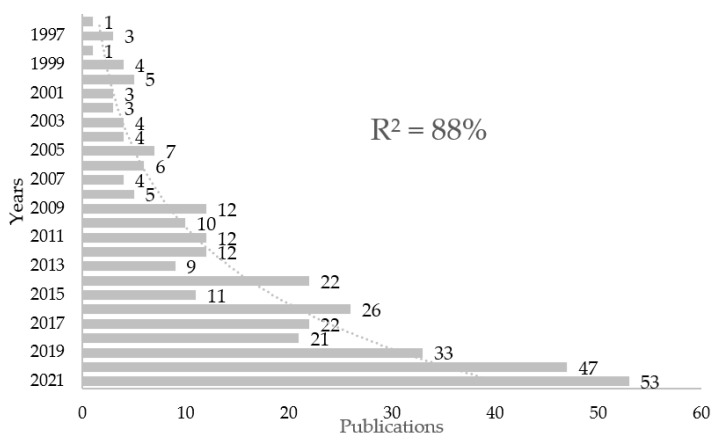
The exponential growth of annual publications on Suicide and Physical Activity.

A continuity of annual publications from 1996 to 2022 was found. The growth of publications in this period was analysed, excluding the year 2022, as it was not completed at the time of the analysis, resulting in an adjusted coefficient of determination with an exponential growth ratio equal to R^2^ = 88% (Figure 1). The most current median of publications was concentrated from 2017 to 2022 (197 publications, 53.5%), with a higher number of publications than in the previous 26 years (171 publications, 46.5%).

### 3.3. Publications Titles

According to the number of documents, Bradford’s core was composed of the 16 most productive journals on the topic. Table 2 shows Bradford’s core journals, with the Journal of Affective Disorder (22 documents) being the journal with the highest number of publications.

Table 3 shows the distribution of journals and documents in the different Bradford areas, according to the number of publications adjusted with a −4.9% error to Bradford’s theoretical series. The core group of journals accumulated 31% of the documents. According to citations, the core group of journals was composed of nine journals that accumulated 33.4% of the citations, Zone I of 26 journals (33.8%), and Zone II of 178 journals (32.9%). The distribution of journals in the Bradford zones, according to the number of citations, was adjusted with a −9.2% error to the Bradford theoretical series (Table 2). Clinical Psychological Science was the most cited journal. Table S1 shows the core journals according to the number of citations.

Thus, the core group of journals was composed of nine journals that accumulated 33.4% of the citations, Zone I of 26 journals (33.8%), and Zone II of 178 journals (32.9%). The distribution of journals in the Bradford zones, according to the number of citations, was adjusted with a −9.2% error to the Bradford theoretical series (Table 3).

### 3.4. Most Prolific and Influential Co-Authors

**Figure 2:**
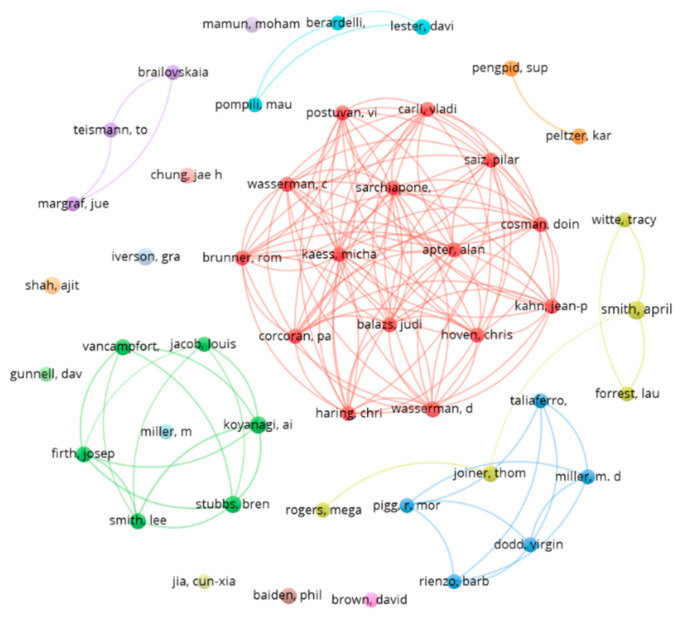
Top 48 most prolific co-authors and their relationships.

When analysing the total number of publications authorship, 1544 co-authors with a distribution between 1–8 documents were found. It was estimated, applying Lotka’s law, that the number of most prolific co-authors should be equal to or less than 39 (square root of 1544). As 16 co-authors were found with four or more papers and 48 co-authors with three or more papers, the latter were considered the most prolific. The 39th most prolific author had three papers. Thus, as displayed in Figure 2, 48 authors with three or more publications were considered the most prolific (analysis: fractionalisation; attraction: 10; repulsion: −2).

Lotka’s law was applied together with the h-index to highlight the most prominent co-authors in the subject area (Table 4). The most demanding Lotka’s law consideration together with the h-index was used to identify the most prominent authors, considering the most prominent the h co-authors with h or more citations among the 16 most productive (16 or more citations and four or more documents).

**Figure 3:**
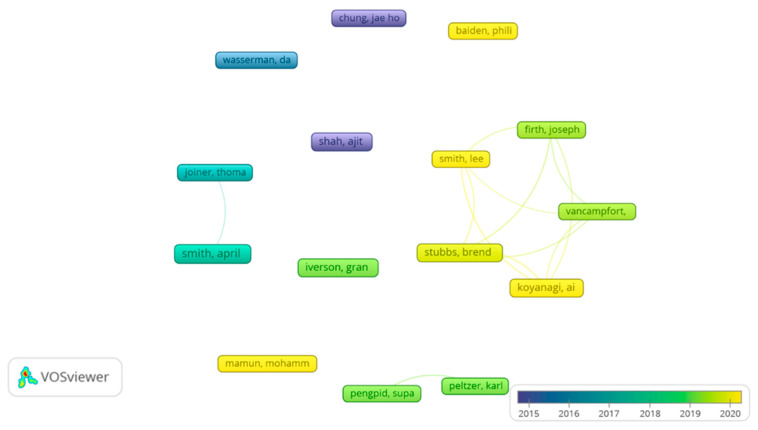
Interrelations between the most prominent co-authors.

Figure 3 shows the interrelations between the most prominent co-authors (analysis: association strength; attraction: 10; repulsion: −2; node size: as a function of the number of publications; colour: as a function of the average number of years of each author’s publications).

### 3.5. Countries/Regions

**Figure 4:**
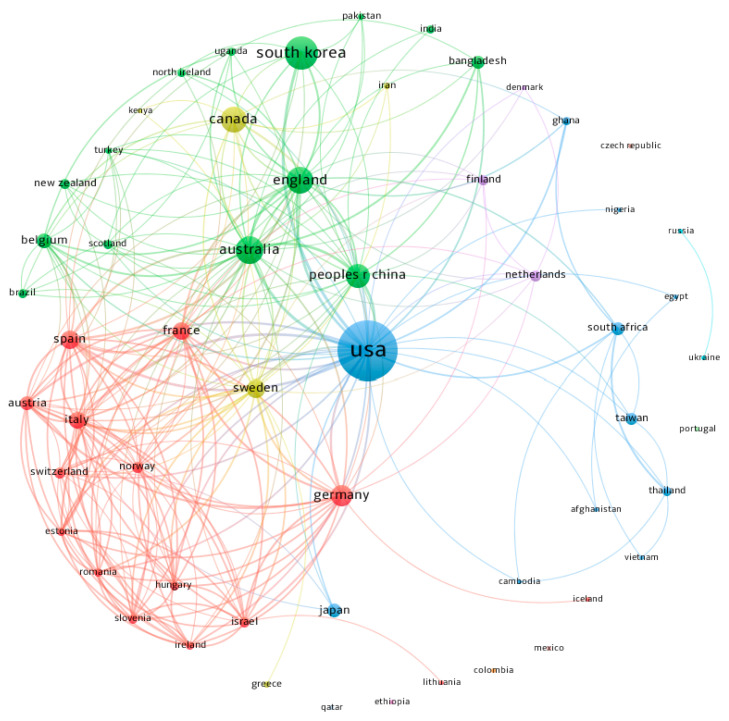
Co-authorship networks by countries/regions.

Among the 56 co-authors countries/regions, the USA was the most productive with 156 of the publications (42.4%), followed by South Korea (48 papers), Australia (35), England (33), and Canada (30). Among the countries/regions with the highest number of citations, again, the USA (4181 citations) was the reference country, followed by Australia (626 citations), Germany (603), South Korea (533), and Canada (522). Figure 4 shows the analysis of the scientific collaborative network between countries/regions (analysis: fractionalization; attraction: 10; repulsion: 0; colour: cluster of relationships between countries; node size: number of publications; thickness of connections: link strength). Once more, the USA was the centre of a global collaborative network, with links to 35 countries, although other countries such as Australia (30) and England (26) also had a large collaborative network.

### 3.6. Most Cited Papers

Table 5 shows the 10 most cited papers and their number of citations. When applying the h-index, 40 articles were found with at least 41 citations (Table S1).

### 3.7. Author Keywords

**Figure 5:**
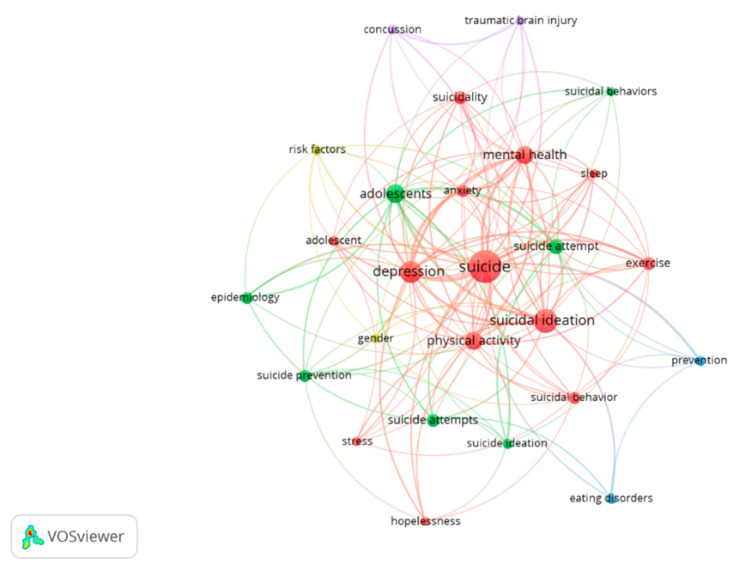
Author keywords.

A total of 778 author keywords were found. Applying Zipf’s law to the frequency analysis of used keywords by co-authors, the 26 words with six or more uses were highlighted (the first number equal to or less than the number estimated by Zipf’s law, 28, square root of 778). The most frequently used terms were: “suicide” (115 occurrences), “suicidal ideation” (57), “depression” (45), “adolescents” (33), and “physical activity” (31). Figure 5 shows the most frequently used author keywords and the relationships between them in the set of articles (analysis: association strength; attraction: 6; repulsion: −2).

## 4. Discussion

The present bibliometric study analyses the properties of 368 documents on suicide and PA published from 1996 to 2021 in the WoS. Finally, 349 papers covering primary research and 19 review articles were found. There has been continuity in the number of publications per year during the analysed period (1997–2022) without any substantial drop in productivity over the time analysed. Analysing the number of publications evolution per year, an exponential growth curve was found, which confirms that the suicide and PA topic is in a period of growing popularity and increasing research interest. This was also confirmed when observing the number of publications from the last five years. Thus, from 2017 to 2021, the total number of documents exceeds the number of publications from the 21 years elapsed between 1996 and 2016. These results are in line with those found in other bibliometric studies related to suicides, “A scientometric analysis of suicide research: 1990–2018” [51], “Research on psychache in suicidal population: a bibliometric and visual analysis of papers published during 1994–2020” [54], “COVID-19 and suicidal behavior: a bibliometric assessment” [62], and “Thirty years of research in suicidology in France: a bibliometric study” [53], all of them reporting a high growth of research interest in suicide and suicidal behaviour.

The analysed documents were catalogued in 70 WoS categories. According to the number of papers published in the journals in every WoS category, the top was Psychiatry, Public Environmental Occupational Health, Psychology Multidisciplinary, Clinical Neurology, Psychology-Clinical, Psychology, Medicine General Internal, Sport Sciences, Neurosciences, and Medicine Legal, all of them with more than 15 documents on the topic Psychiatry was the more prominent category with 155 documents, with 42% of the papers related to this topic, in line with the 46% found by Astraud et al. [53]. Far behind were the second category of Public Environmental Occupational Health (54 documents) and the third one, Psychology Multidisciplinary (44 publications), in productivity terms. This increased interest related to the Psychiatry category was also reflected in the most used author keywords (depression, anxiety, mental health, stress), in line with other bibliometrics on suicide [54,62] and most cited papers. The three papers with the highest number of citations belonged to this thematic category: “Increases in depressive symptoms, suicide-related outcomes, and suicide rates among U.S. adolescents after 2010 and links to increased new media screen time” [63], an article based on survey data from over 500,000 U.S. adolescents, relating adolescents’ new media screen time to increased depression, depressive symptoms and increased suicides; “Life satisfaction and suicide: a 20-year follow-up study”, a research in which more than 29,000 adults were followed for 20 years, concluding that life dissatisfaction was a risk factor for suicide, and those who reported life dissatisfaction at baseline and 6 years later had a higher risk of suicide (hazard ratio = 6.84, 95% Confidence Interval = 1.99–23.50) compared to those who repeatedly reported satisfaction [12]; and “Physical activity and personal characteristics associated with depression and suicide in American College Men” [64], a classical study from 1994, in which Harvard ex-students were followed over a long period, concluding that depression rates were lower in active and athletic subjects and that there was no relationship between previous PA and suicide rate, unlike among smokers and people with personality traits that predicted higher rates of depression. The first article related to a category other than Psychiatry was the fourth most cited: “Aggression, substance use, and suicidal behaviours in high-school-students” [65], an article related to the Public Environmental Occupational Health category.

The Journal of Affective Disorders was the most productive journal with 22 publications, the most productive journal in the Psychiatric and Clinical Neurology categories. This journal could be one of the preferred options for researchers to publish their research articles in one of these categories. Although it was not the journal with the most publications in the bibliometrics found on suicide, it was found to be among the most productive in all of them, being third in the bibliometrics “Thirty Years of Publications in Suicidology” [53] and the second one in “Research on Psychache in Suicidal Population” [54]. Public Environmental Occupational Health was the second most published journal and the first in the category Public Environmental Occupational Health, an open-access journal from MDPI that was also among the top 10 most productive journals in the bibliometrics on “COVID-19 and suicidal behaviour” by Grover et al. [62], being an interesting multidisciplinary journal for researchers who intend to publish their research on more open suicide topics. Psychiatry Research (Elsevier) and Suicide and Life-Threatening Behavior (Wiley) were the other two journals more interested in the topic, the second one the most productive in the Psychology Multidisciplinary category and the one containing the most publications in the bibliometrics paper “Thirty Years of Publications in Suicidology” [53]. However, the journal with the highest number of citations was Clinical Psychological Science, and despite only presenting a single article, it was the most cited of all the analysed journals [63]. 

Despite being a global problem, only 56 countries were the origin of the documents found. Cultural considerations and social taboos, suicide stigmatisation or the lack of investment in prevention strategies may be some of the reasons for the lack of involvement in suicide and PA in many countries [51,66,67,68]. The number of countries involved in suicide and PA research was slightly lower than those found in other topics, such as the analysis of research on diabetes, depression, or suicide, with 83 countries involved [69]; the paper on COVID-19 and suicide, with 78 countries [62]; or the one conducted by Cai et al. [50] on suicide research between 1998 and 2018, with 117 countries involved. The USA’s leadership was a common denominator in all research, as it was the most productive country in suicide research and the centre of research networks around the world, collaborating with co-authors from 35 countries. In our research, 42.4% of publications had a co-author from the USA, a percentage close to the 30–40% found in other studies [51,53,62]. Other countries forming strong research groups include England, South Korea, Canada, and multi-national research groups from European countries. The same was reflected in the origin of the most prolific co-authors; April Smith (University of Miami) was the most productive from the USA as well as other co-authors such as Grant L. Iverson, Thomas Joiner, or Philip Baiden, with other prolific co-authors from England (Stubbs, Shah, and Smith), South Korea (Chung), or Spain (Koyanagi), all of them very active and highly cited co-authors in the field.

Among the practical implications of this bibliometric study is to reveal the growing state of interest of the state of interest in suicide- and PA-related research on the part of publishers, journals, and editors as well as the base of researchers behind the publications on this subject, ensuring a firm basis on which to increase knowledge in this area and research ensuring that the documents on the subject could have a significant follow-up by them and generate publications with a high number of readerships and high impact. Moreover, it provides information to researchers and research groups on research trends in this topic as well as represents a potential opportunity to know researchers and facilitate collaboration, partnerships, and networks. The main limitation is that, despite using one of the most complete databases, the WoS, a publication bias may have occurred due to not using other sources such as PubMed or Scopus.

## 5. Conclusions

Suicide and PA annual publications showed an exponential growth trend in recent years, including many researchers, journals, and publishers interested in the topic. The USA was the most productive country with 156 publications (42.4%), and it was the most cited (4181 citations). It also was the centre of a collaborative network, with links to 35 countries. The most prolific author (eight publications), April Smith, and the most cited author (513 citations), Thomas Joiner, also developed their research in this country. The Psychiatry category, with 155 papers, had the highest number of publications, and The Journal of Affective Disorders, from Elsevier, had the highest number of papers published in this category.

## Figures and Tables

**Table 1:** Web of Science Top 10 most important categories on Suicide and Physical Activity papers.

WoS Categories	Number	Main Journals Abbrev. (Number)	Publisher
Psychiatry	155	J Affect Disorders (22)	Elsevier
Public Environmental Occupational Health	54	Int J Env Res Pub He (15)	MDPI
Psychology Multidisciplinary	44	Suicide Life-Threat (9)	Wiley
Clinical Neurology	37	J Affect Disorders (22)	Elsevier
Psychology Clinical	31	J Clin Psychol (5)	Wiley
Psychology	25	Arch Suicide Res (4) Int Psychogeriatr (4)	Taylor & Francis Cambridge Univ
Medicine General Internal	21	BMJ Open (3) J Korean Med Sci (3)	BMJ Publishing Korean cad
Sport Sciences	18	Scan J Med Sci Spor (3)	Wiley
Neurosciences	17	Front Neurol (3)	Frontiers Media
Medicine Legal	15	Forensic Sci Int (3) Int J Legal Med (3) J Forensic Sci (3)	Elsevier Springer Wiley

**Table 2:** Bradford Core Journals.

Bradford’s Zone	Journals (Publisher)	Art.	%Art.	%Acc.	Cites	Q.	%O.A.
Core	Journal of Affective Disorders (Elsevier)	22	6	6	339	Q2	6
	International Journal of Environmental Research and Public Health (MDPI)	11	3	9	24	Q1	95
	Psychiatry Research (Elsevier)	10	3	12	189	Q2	4.7
	Suicide and Life-Threatening Behavior (Wiley)	9	2	14	115	Q1	6.3
	Frontiers in Psychiatry (Frontiers Media)	8	2	16	88	Q2	99.3
	Bmc Psychiatry (BMC)	7	2	18	83	Q2	100
	Plos One (Public Library Science)	6	2	20	130	Q2	99.6
	Social Psychiatry and Psychiatric Epidemiology (Springer Heidelberg)	5	1	21	54	Q1	30.9
	BMC Public Health (BMC)	5	1	23	125	Q2	99.6
	Canadian Journal of Psychiatry-Revue Canadienne de Psychiatrie (SAGE)	5	1	24	37	Q2	24
	Journal of Clinical Psychology (Wiley)	5	1	26	148	Q3	9.7
	Acta Psychiatrica Scandinavica (Wiley)	4	1	27	385	Q1	28.7
	Aggression and Violent Behavior (Elsevier)	4	1	28	20	Q1	4.3
	Archives of Suicide Research (Taylor & Francis)	4	1	29	44	Q2	7.1
	Crisis—The Journal of Crisis Intervention and Suicide Prevention (Hogrefe)	4	1	30	37	Q2	3
	International Psychogeriatrics (Cambridge University Press)	4	1	31	41	Q1	18.3

Art. (articles); %Art. (percentage of articles); %Acc. (percentage of accumulated articles); Q. (journal citation report quartile); %O.A. (percentage of open access).

**Table 3:** Bradford’s zones and their number of journals, based on the number of publications.

**According to the Number of Documents**												
Zone	Journals (%)		Papers (%)		Acc. n° journals (%)		Acc. papers n° (%)		Bradford multipliers		Journals (theoretical series)	
CORE	16	(8%)	113	(31%)	16	(8%	113	(31%)			n0	16
Zone 1	43	(20%)	101	(27%)	59	(28%)	214	(58%)	2.69		n1	50
Zone 2	154	(72%)	154	(42%)	213	(100%)	368	(100%)	3.58		n2	157
Total	213	100%	368	100%					Mean	3.1		223
											% Error	−4.9%
**According to the Number of Citations**												
Zone	Journals (%)		Papers (%)		Citations (%)		Acc. n° citations (%)		Bradford multipliers		Journals (theoretical series)	
CORE	9	(4%)	48	(13%)	2452	(33%)	2452	(33%)			n0	9
Zone 1	26	(12%)	68	(18%)	2480	(34%)	4932	(67%)	2.89		n1	50
Zone 2	178	(84%)	252	(68%)	2416	(33%)	7348	(100%)	3.71		n2	174
Total	213	100%	368	100%	7348	(100%)			Mean	3.3		233
											% Error	−9.2%

n° (number); % (percentage); Acc. (accumulated).

**Table 4:** Most prominent co-authors on Suicide and Physical Activity.

Co-Authors	Affiliation/Countries-Regions	Papers	Citations
Smith, A.	Miami University/United States of America	8	156
Stubbs, B	King’s College London/England	6	153
Koyanagi, A.	Catalan Institution for Research and Advanced Studies/Spain	6	70
Shah, A.	University of Central Lancashire/England	6	40
Iverson, G.	Harvard Medical School/United States of America	5	107
Joiner, T.	Florida State University/United States of America	4	513
Wasserman, D.	National Institute for Health, Migration and Poverty/Italy	4	225
Firth, J.	University of Melbourne/Australia	4	131
Vancampfort, D.	Catholic University of Leuven/Belgium	4	121
Chung, J.	Seoul National University/South Korea	4	78
Baiden, P.	University of Texas Arlington/United States of America	4	56
Peltzer, K.	University of Limpopo/South Africa	4	48
Pengpid, S.	Mahidol University/Thailand	4	48
Smith, L.	Anglia Ruskin University/England	4	46
Mamun, M.	Centre for Health Innovation, Networking, Training, Action, and Research/Bangladesh	4	38

**Table 5:** Most cited documents in Suicide and Physical Activity.

Papers	Journal Abbrev.	Citations
Increases in Depressive Symptoms, Suicide-Related Outcomes, and Suicide Rates Among U.S. Adolescents after 2010 and Links to Increased New Media Screen Time	Clin Psychol Sci	422
Life Satisfaction and Suicide: A 20-Year Follow-Up Study	Am J Psychiat	281
Physical Activity and Personal Characteristics Associated with Depression and Suicide in American College Men	Acta Psychiat Scand	281
Aggression, Substance Use, and Suicidal Behaviours in High-School-Students	Am J Public Health	254
Chronic Traumatic Encephalopathy, Suicides and Parasuicides in Professional American Athletes the Role of the Forensic Pathologist	Am J Foren Med Path	149
Depression And Suicide Ideation Among Students Accessing Campus Health Care	Am J Orthopsychiat	134
Risk of Completed Suicide after Bariatric Surgery: A Systematic Review	Obes Rev	125
Sports Participation as a Protective Factor Against Depression and Suicidal Ideation in Adolescents as Mediated by Self-Esteem and Social Support	J Dev Behav Pediatr	120
Cigarette Smoking and Suicide: A Prospective Study Of 300,000 Male Active-Duty Army Soldiers	Am J Epidemiol	117

Abbrev. (abbreviation).

## Data Availability

Datasets will be available upon reasonable request to the corresponding author.

## Supplementary Materials

The following supporting information can be downloaded at: https://www.mdpi.com/article/10.3390/ijerph192416413/s1, Table S1: Documents more cited in Suicide and Physical Activity.
