# Supplementary material for: Mapping the Scientific Research on Suicide and Physical Activity: A Bibliometric Analysis
Source: Int J Environ Res Public Health. 2022 Dec 7;19(24):16413. doi: 10.3390/ijerph192416413 (PMC9778543; doi:10.3390/ijerph192416413)
Supplement: Supplementary file 1 [file ijerph-19-16413-s001.zip › ijerph-2043493-supplementary.pdf]

**Supplementary Material**

**Table S1.** Documents more cited in Suicide and Physical Activity.

| Manuscript | Journal Abbrev. | Citations |
|---|---|---|
| Increases in Depressive Symptoms, Suicide-Related Outcomes, and Suicide Rates Among U.S. Adolescents After 2010 and Links to Increased New Media Screen Time | Clin Psychol Sci | 422 |
| Life Satisfaction and Suicide: A 20-Year Follow-Up Study | Am J Psychiat | 281 |
| Physical Activity and Personal Characteristics Associated with Depression and Suicide in American College Men | Acta Psychiat Scand | 281 |
| Aggression, Substance Use, and Suicidal Behaviours in High-School-Students | Am J Public Health | 254 |
| Chronic Traumatic Encephalopathy, Suicides and Parasuicides in Professional American Athletes the Role of The Forensic Pathologist | Am J Foren Med Path | 149 |
| Depression and Suicide Ideation among Students Accessing Campus Health Care | Am J Orthopsychiat | 134 |
| Risk of Completed Suicide After Bariatric Surgery: A Systematic Review | Obes Rev | 125 |
| Sports Participation as A Protective Factor Against Depression and Suicidal Ideation in Adolescents as Mediated by Self-Esteem and Social Support | J Dev Behav Pediatr | 120 |
| Cigarette Smoking and Suicide: A Prospective Study of 300,000 Male Active-Duty Army Soldiers | Am J Epidemiol | 117 |
| Cigarettes and Suicide: A Prospective Study of 50 000 Men | Am J Public Health | 112 |
| Suicidal Ideation and Attempts in Adolescents: Associations with Depression and Six Domains of Self-Esteem | J Adolescence | 107 |
| Hours of Sleep in Adolescents and its Association with Anxiety, Emotional Concerns, and Suicidal Ideation | Sleep Med | 99 |
| A Newly Identified Group of Adolescents at Invisible Risk for Psychopathology and Suicidal Behaviour: Findings from the SEYLE Study | World Psychiatry | 98 |
| Declining Autopsy Rates and Suicide Misclassification: A Cross-National Analysis of 35 Countries | Arch Gen Psychiat | 95 |
| Lipid-Lowering Drugs and the Risk of Depression and Suicidal Behaviour | Arch Intern Med | 91 |
| Associations Between Cyberbullying and School Bullying Victimization and Suicidal Ideation, Plans and Attempts Among Canadian Schoolchildren | Plos One | 88 |
| Physical Activity and Suicidal Ideation: A Systematic Review and Meta-Analysis | J Affect Disorders | 85 |
| Body Mass Index and Risk of Suicide among Men | Arch Intern Med | 82 |
| High School Youth and Suicide Risk: Exploring Protection Afforded Through Physical Activity and Sport Participation | J School Health | 82 |
| Feasibility and Utility of Positive Psychology Exercises for Suicidal Inpatients | Gen Hosp Psychiat | 80 |
| Patterns Of Suicide by Occupation in England and Wales: 2001-2005 | Brit J Psychiat | 80 |
| Exercise Caution: Over-Exercise is Associated with Suicidality among Individuals with Disordered Eating | Psychiat Res | 77 |
| On Buckeyes, Gators, Super Bowl Sunday, and the Miracle on Ice: Pulling Together is Associated with Lower Suicide Rates | J Soc Clin Psychol | 76 |
| Physical Activity, Sports Participation, And Suicidal Behaviour Among College Students | Med Sci Sport Exer | 74 |
| A Systematic Review of Validated Methods for Identifying Suicide or Suicidal Ideation Using Administrative or Claims Data | Pharmacoepidem Dr S | 73 |
| Suicidal Thoughts Among Adolescents - An Intercultural Approach | Adolescence | 73 |
| Associations Between Physical Activity and Reduced Rates of Hopelessness, Depression, and Suicidal Behavior Among College Students | J Am Coll Health | 68 |
| Chronic Traumatic Encephalopathy and Risk of Suicide in Former Athletes | Brit J Sport Med | 67 |
| Cardiovascular Disease Risk Factors in Relation to Suicide Mortality in Asia: Prospective Cohort Study of Over One Million Korean Men and Women | Eur Heart J | 57 |
| The Impact of Experiential Exercises on Communication and Relational Skills in A Suicide Prevention Gatekeeper-Training Program for College Resident Advisors | J Am Coll Health | 51 |
| A New Factor in Youth Suicide: The Relative Age Effect | Can J Psychiat | 50 |
| Physical Activity, Sadness, and Suicidality in Bullied US Adolescents | J Am Acad Child Psy | 49 |

| | | |
|---|---|---|
| The Impact of Exercise on Suicide Risk: Examining Pathways Through Depression, PTSD, and Sleep in An Inpatient Sample of Veterans | Suicide Life-Threat | 47 |
| Resilience As a Focus of Suicide Research and Prevention | Acta Psychiat Scand | 46 |
| Why Alternative Teenagers Self-Harm: Exploring the Link Between Non-Suicidal Self-Injury, Attempted Suicide and Adolescent Identity | Bmc Psychiatry | 45 |
| Self-Reported Happiness in Life and Suicide in Ensuing 20 Years | Soc Psych Psych Epid | 45 |
| Suicide In National Collegiate Athletic Association (NCAA) Athletes: A 9-Year Analysis of The NCAA Resolutions Database | Sports Health | 44 |
| Associations Between Eating Disorder Symptoms and Suicidal Ideation Through Thwarted Belongingness and Perceived Burdensomeness among Eating Disorder Patients | J Affect Disorders | 43 |
| Associations Between Lifestyle Factors, Working Environment, Depressive Symptoms and Suicidal Ideation: A Large-Scale Study in Japan | Ind Health | 43 |
| Injured Athletes and The Risk of Suicide | J Athl Training | 43 |
